# Long-term cross sectoral care and case management for people with severe multiple sclerosis and their caregivers – impact on dimensions of life

**DOI:** 10.1186/s12913-026-14177-y

**Published:** 2026-03-14

**Authors:** Anne Müller, Veronika Dunkl, Wiebke Müller, Martin Hellmich, Peter Löcherbach, Raymond Voltz, Kim Dillen, Yasemin Goereci, Clemens Warnke, Heidrun Golla

**Affiliations:** 1 Department of Palliative Medicine, Faculty of Medicine and University Hospital, University of Cologne, Cologne, Germany; 2 Institute of Medical Statistics and Computational Biology (IMSB), Faculty of Medicine and University Hospital, University of Cologne, Cologne, Germany; 3 Department of Medical Statistics, University Medical Center Göttingen, Göttingen, Germany; 4 German Society of Care and Case Management E.V. (DGCC), Münster, Germany; 5 Center for Integrated Oncology Aachen Bonn Cologne Düsseldorf (CIO ABCD), University of Cologne, Cologne, Germany; 6 Center for Health Services Research (ZVFK), University of Cologne, Cologne, Germany; 7 Department of Neurology, Faculty of Medicine and University Hospital, University of Cologne, Cologne, Germany; 8 Department of Neurology, University Hospital Gießen and Marburg, Philipps University Marburg, Marburg, Germany; 9 Department of Palliative Medicine, University Medical Center Göttingen, Göttingen, Germany; 10 Institute for Health Economics and Clinical Epidemiology (IGKE), Faculty of Medicine and University Hospital, University of Cologne, Cologne, Germany; 11 Department of Neurology, Klinikum Köln, Cologne, Germany; 12 Clinical Trials Centre Cologne (CTCC), Faculty of Medicine and University Hospital, University of Cologne, Cologne, Germany; 13 NeuroMed Campus, MedCampus Hohenlind, Cologne, Germany; 14 German Multiple Sclerosis Society Federal Association (DMSG), Hannover, Germany; 15 Department of Neurology, Heilig Geist-Krankenhaus Köln, Cologne, Germany; 16 Academic Teaching Practice, University of Cologne, Cologne, Germany; 17 Department of Neurology, Klinikum Köln-Merheim, Cologne, Germany

**Keywords:** Health care, Social care, Self-sufficiency, Social participation, Autonomy, Physical health, Mental health

## Abstract

**Background:**

Multiple sclerosis is associated with complex individual disease trajectories with a high symptom burden affecting physical, psychological and social life, accompanied by fractured biographies and increased suicide rates. In this complex situation coordinating and providing necessary health and social services and treatments by themselves is often perceived as unmanageable. Care and case management (CCM) aims to ensure optimal care in all dimensions of life. The aim of this sub-study of the COCOS-MS (Communication, coordination and security for people with severe Multiple Sclerosis) trial was to evaluate the CCM intervention at the individual and overall level.

**Methods:**

The analysis of the long-term cross-sectoral CCM intervention included: descriptive statistical analysis of the individually defined goals and actions taken together with the CCM and the statistical evaluation of the evolution of unmet needs of people with severe MS (PwsMS) and caregivers considering physical health, mental health, self-sufficiency, social situation and participation utilizing the sign test (α < 0.05) and cross tables. The data were analyzed in accordance with the modified intention-to-treat principle.

**Results:**

Compliant to protocol, a total of 40 PwsMS were randomized to the intervention group; 18 caregivers of these PwsMS also participated. In 80% the individually set goals were realized during the CCM intervention. They were mostly related to healthcare, organizational issues or general aspects like: receiving information). The participants benefited from the CCM in all assessed dimensions, which was especially reflected in a significant reduction of unmet needs in relation to: MS related physical health (*p* = 0.039), medical, nursing and therapeutic care (*p* = 0.008), and increased autonomy regarding the pursuit of hobbies and interests (*p* = 0.002). Across 30 sub-domains, on average 6.3 improvements and 1.6 deteriorations per sub-domains were recorded on PwsMSs’ side.

**Conclusions:**

The CCM is a worthwhile approach to the comprehensive networking of health and social structures. It helps PwsMS to manage their disease and its associated problems more effectively and broadly. Thereby, they gain resources for their life outside their illness. Improved self-sufficiency and social participation lead to a consolidated identity. This could increase feelings of dignity and counteract life-denying thoughts.

**Trial registration:**

German Register for Clinical Studies (DRKS) (DRKS00022771, 11^th^ January 2021).

**Supplementary Information:**

The online version contains supplementary material available at 10.1186/s12913-026-14177-y.

## Introduction

Multiple sclerosis (MS) is the most prevalent chronic, inflammatory, and degenerative neurological disorder of the central nervous system (CNS), accompanied by significant social and economic impact [1], [2–4]. Individual disease trajectories show a high degree of heterogeneity, manifested in a range of symptoms with physical and/or mental impairments. Monitoring disease-specific therapy, organizing daily life, care and treatment throughout the disease trajectory with its changing needs, up to palliative and hospice care, and attempting to regain life roles are just some of the challenges faced by people with severe MS (PwsMS) and their caregivers. [4–8]. Coordinating and providing the necessary health and social care structures and treatments is often perceived as stressful and unmanageable by PwsMS and their caregivers, due to the high level of bureaucracy and complexity of the German health and social care system, with each institution working independently [5, 9–12].

Case Management (CM) is a desirable approach supporting patients and their caregivers during their patient journey [12, 13] It may have positive effects on symptom burden and unmet needs in patients with chronic diseases and complex requirements. [12, 14, 15]. However, reflections and studies on CM rarely include patients with complex palliative care needs and/or patients with complex and severe neurological long-term conditions such as MS [11, 16–19], who are especially restricted in managing (everyday) life due to physical, mental, cognitive, neuropsychological and/or communicative impairments.

The impact of a long-term cross-sectoral care and case management (CCM) approach for PwsMS and their caregivers (family members or closely related persons, directly involved in patient care) has not yet been evaluated. This discrepancy can be attributed to the absence of a structured CCM framework for this patient population. The inclusion of PwsMS in clinical trials is particularly rare, as they are difficult to reach because they are cared for privately rather than in the existing health and social care system [20–23].

Consequently, we exploratorily tested a CCM intervention for this specific patient population within the COCOS-MS phase II clinical trial (Communication, coordination and security for people with severe Multiple Sclerosis) [24]. For the PwsMS and their caregivers investigated in the intervention group, a study specific CCM manual was developed [25]. With help of this CCM manual comprehensive dimensions of life were individually mapped including physical and mental health, social life, financial-, everyday- and bureaucratic issues. The domains of life that were investigated were chosen and composed of a variety of validated and established guidelines and questionnaires [25–35]. The CCM manual aims to facilitate the identification, implementation, and coordination of necessary treatments and services. The case managers, operate on an individual level, with the aim of improving problems, resources and resulting unmet needs of PwsMS and their caregivers. On the superordinate operational level addressed by the care management, the objective was to coordinate and manage health and social care services, implement a connection, transfer information between healthcare specialists, as well as identify gaps in care.

The objective of this single-arm trial as part of the explorative randomized clinical trial was to ascertain how identified problems, resources and resulting unmet needs of PwsMS and their caregivers in the intervention group changed over the CCM intervention period of twelve months. In addition, we examined the extent of individual goal achievement that had been agreed on with PwsMS and caregivers. On the superordinate care management level, we aimed to develop a portfolio of cooperation partners related to this particular PwsMS population in the region of Cologne, Germany.

## Methods

### Trial design

The COCOS-MS trial is a randomized controlled phase II clinical trial with parallel arms testing a complex intervention with an embedded qualitative study part (mixed-method design). In this sub-study we focused on the analysis of the complex CCM intervention delivered in the intervention group according to the study specific CCM manual [24, 25, 36, 37].

### Participants

In the absence of a universally accepted definition of ‘severe MS’, we used a three-pronged approach to identify people with the most severe forms of the disease, in consultation with MS medical specialists. This involved categorizing individuals based on their expanded disability status scale (EDSS) [38], age, and the immunotherapeutic treatment options they were receiving. To be included in subgroup 1, participants were required to meet the following criteria: (1) they had to have highly active MS, (2) they had to be treated with an escalating immunotherapeutic agent, (3) they had to have an EDSS of at least 5, and/or (4) they had to be aged 50 years or older at baseline. Subgroup 2 was defined as patients with primary or secondary chronic progressive MS, aged ≥18 years, and either (subgroup 2a) moderate disability (EDSS 4–7) with no immunotherapeutic treatment option or (subgroup 2b) severe disability with an EDSS > 7. All participants were required to reside in the administrative district of Cologne encompassing rural and urban regions and be proficient in the German language, or have a legal representative who was proficient in the German language. Patients needed to be able to give full written informed consent. If the patient was unable to give full written informed consent, a legal representative with full command of the German language and able to give full written informed consent could act on behalf of the patient. The primary responsibility of the caregiver was to provide care for the patient, aged ≥18 years, to be proficient in the German language and able to provide written informed consent. PwsMS were permitted to participate in the study regardless of the participation of a caregiver [24, 37].

The CCM intervention was delivered, according to the study protocol, face-to-face at the current location of the PwsMS and caregiver which could e.g. be at home, in a nursing home, rehabilitation center or hospital, online or by telephone [24, 37].

### CCM intervention

**Fig. 1:**
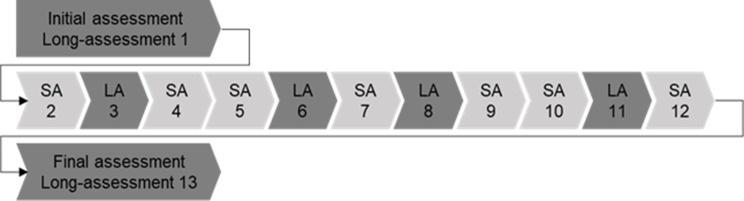
Assessment-flow of the 12 months CCM intervention, SA = short-assessment, LA = long-assessment

PwsMS randomized to the intervention group of the COCOS-MS trial received CCM in addition to standard care. The staff was trained and certified by the ‘Deutsche Gesellschaft für Care and Case Management (DGCC)’. Using the study-specific CCM manual, care and case managers individually tailored their intervention to study participants [36]. In order to ensure comprehensive care (management), the CCM jointly evaluated with study participants their problems, resources and resulting unmet needs across all dimensions of life. A comprehensive patient and caregiver assessment was carried out by the CCM at the start of the CCM intervention (initial assessment = “long-assessment 1”) and every three months thereafter (long-assessments 3, 6, 8, 11, 13, where “long-assessment 13” is the final assessment) (see Fig. 1); the assessment comprised the following dimensions: physical health, mental health, self-sufficiency and social situation and participation. In the intervening months, abbreviated assessments in the mentioned dimensions (short-assessments 2, 4, 5, 7, 9, 10, 12) were carried out.

**Fig. 2:**
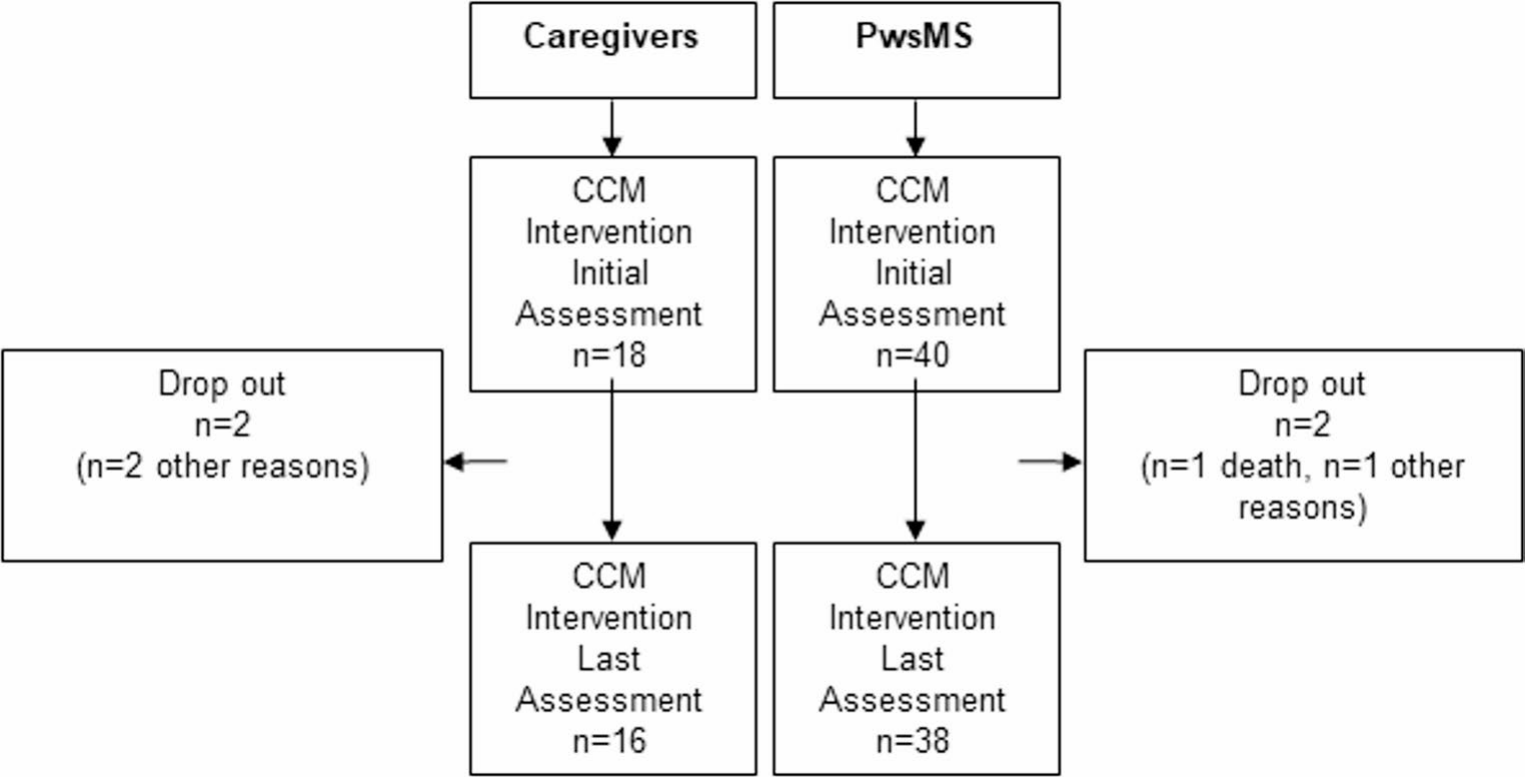
Flow chart - recruitment and adherence in the intervention group of the COCOS-MS study. MOREcare criteria [41] for drop outs: attrition due to death, due to illness, due to random (other reasons)

The care and case managers documented each patient and caregiver contact as well as contacts with healthcare specialists, in the structured CCM manual. The documentation of the assessed dimensions of life (see Table 1) was based on a Likert scale (see Table 2) for each item, which was rated by the study participants and documented by the care and case managers; additional free text comments were possible.

**Table 1:** Dimensions of life and subdimensions assessed and analyzed in this manuscript

Physical Health
• MS specific (e.g. pain, progress of MS, spasticity) • Other physical illness (e.g. cardiovascular) • Medication (e.g. management of immune modulatory therapy)
**Mental health**
• Psychological situation (e.g. fatigue, depression, autonomy, personality change)
**Self-sufficiency**
• Self-care patient: nursing, household and organization (e.g. showering, coordinating appointments)
• Medical, nursing, therapeutic care • General practitioner care • Neurological care, other specialist care • Nursing service • Physiotherapy, occupational therapy, speech therapy, psychotherapy • Information about MS (course, therapy, self-help offers)
• Mobility (e.g. walking, access to transportation)
• Housing (e.g. access to residence, accessibility of bathroom)
• Work and employment (e.g. work ability, voluntary work)
• Financial and social legal situation (e.g. social insurance carriers, debts, financial security)
**• Social situation and participation**
• Communication (e.g. use of mobile phone, access to information)
• Social integration, family and friendship involvement, role as e.g. partner, child, parent, friend
• Pursuing hobbies/interests, leisure time activities (e.g. sufficient leisure time, attending cultural events)
• Cultural/social/political participation (e.g. participation in neighborhood)
• Spiritual/pastoral care, religion (e.g. contact with spiritual/pastoral care)

**Table 2:** Likert scale for resources, problems and needs

Problem 0 = no problem 1 = moderate problem 2 = significant problem 3 = big problem 4 = very big problem	Resource 0 = no resource 1 = scarce resource 2 = sufficient resource 3 = good resource 4 = very good resource	Need 0 = no need 1 = need for monitoring 2 = need for action 3 = need for intervention 4 = crisis intervention

Following the initial CCM assessment (long-assessment 1) in which problems, resources and resulting unmet needs were assessed in the different dimensions of life (physical health, mental health, self-sufficiency, and social situation and participation) a care plan with defined goals was developed by the CCM. It comprised specific, well defined actions in order to achieve the goals; 36 different goals and 125 actions were provided for selection by the CCM manual. Following each comprehensive assessment, the care plan was adapted according to changing resources, problems and resulting unmet needs. This was achieved through an examination and analysis of all dimensions of life. New goals and actions were consistently incorporated and evaluated in consultation with patients and caregivers. In the final assessment, CCM and study participants jointly evaluated which goals, and to what extend, could be achieved. The evaluation was categorized as follows: not achieved, barely achieved, partially achieved, achieved, and more than achieved.

Through the continuous work throughout the CCM process, a network of regional cooperation partners who proved to be competent and available to this group of PwsMS with their specific needs was established and summarized in a portfolio. At the end of the CCM process, this information was individually tailored and made available to the study participants.

### Sample size and randomization

PwsMS were recruited in rural and urban districts of Cologne (4.54 million inhabitants) under the direction of the Departments of Palliative Medicine and Neurology at Cologne University Hospital, through outpatient and inpatient neurological institutions, the national German Multiple Sclerosis Society (DMSG) register, and the local DMSG group in Cologne [37]. Study patients (*n* = 80) were randomly assigned to either the intervention or the control group (allocation ratio 1:1). The sample size calculation was conducted for the COCOS-MS trial. Randomization was done by an internet-based tool (ALEA; FormsVision BV, Abcoude, Netherlands) and stratified by subgroup (1, 2a, 2b). We aimed and fulfilled an equal enrolment (1:1:1) in the three subgroups (1, 2a, 2b). The sample which we investigated here with respect to the CCM intervention represents the intervention group of the COCOS-MS trial which consisted of 40 PwsMS and 18 caregivers.

### Statistical methods

The sign test is an exact non-parametric method used to determine whether paired data show any imbalance in positive and negative differences. It can be applied when data are not normally distributed [39], in particular to ordinal data. As our data is not normally distributed, the sign test (α < 0.05) was employed for the paired data setting with the ordinal scaled items of problems, resources, and resulting unmet needs. The care plan, care coordination, and sociodemographic characteristics were subjected to descriptive statistical analysis (median with minimum and maximum, mean± standard deviation). All analyses were conducted using the software IBM SPSS Statistics 29®. No adjustments were made for multiple testing – in order not to obscure possibly meaningful patterns in our data. The modified intention-to-treat (mITT) dataset includes all trial subjects (PwsMS and caregivers, if any) enrolled in the trial and randomized with a valid baseline assessment (T0) and at least one follow-up assessment. The comments field for individual goals in the care plan were qualitatively analyzed according to a thematical analysis [40].

## Results

### Recruitment

Between April 2021 and January 2022, participants were recruited for the COCOS-MS study. The CCM intervention was implemented between April 2021 and January 2023 for the duration of twelve months for each participant.

### Participant flow of the intervention group

Of the 40 PwsMS randomized to the intervention group (22 female, 18 male) the subgroup allocation was as follows: subgroup 1, subgroup 2a. subgroup 2b, 14:13:13. PwsMS were accompanied by a caregiver also participating in the study in 18 cases. A total of 38 PwsMS and 16 caregivers completed the 12-month intervention. One PwsMS died during the intervention phase (data included), one PwsMS and one caregiver could no longer be reached or did not wish to participate further. One caregiver terminated the relationship with the patient, and the patient declined further contact with the CCM Fig. 2.

### Sociodemographic data

The average age of PwsMS was 53.5 years (min. 32, max. 80 years), the average age of caregivers was 56.1 years (min. 39, max. 81 years). At the time of enrollment in the study, the EDSS of PwsMS studied here ranged from min. 5 to max. 9 (mean: 6.7). Disease duration ranged from min. 2 to max. 44 years (mean: 18.1). Upon enrollment in the study, the degree of disability (in German: *Grad der Behinderung* (GdB) rates the degree of physical, mental/emotional impairments on a scale from 20 to 100 and determines eligibility for legal rights and compensations) was 90 on average (median: 100). Sixteen (40%) of the PwsMS had advance directives, with 12 of them having a patient decree, too. For details of sociodemographic data see also Table 3.

**Table 3:** Sociodemographic characteristics of the CCM intervention group

	PwsMS				Caregiver
	Total (*n* = 40)	Subgroup 1 (*n* = 14, 35%)	Subgroup 2a (*n* = 13, 32.5%)	Subgroup 2b (*n* = 13, 32.5%)	Total (*n* = 18)
Age in years, mean (SD)	53.5 (10.6)	46.6 (8.9)	58.8 (10.1)	55.5 (9.4)	56.1 (10.3)
Sex, n (%) - female - male	22 (55) 18 (45)	5 (35.7) 9 (64.3)	10 (76.9) 3 (23.1)	7 (53.8) 6 (46.2)	10 (55.6) 8 (44.4)
EDSS, mean (SD) - at first assessment - at the end of the intervention	6.4 (1) 6.3 (1.3)	6.3 (1) 5.9 (1.5)	6.0 (0.5) 6 (0.9)	8 (0) 7.8 (0.5)	-
Duration of the disease in years, mean (SD)	18.6 (10.8)	17	18.8	19.9	-
Level of care, n (mean) - at first assessment - at the end of the intervention	30 (3) 35 (3)	10 (3) 13 (3)	9 (3) 12 (2)	11 (2) 10 (4)	-
GdB (median) - at initial assessment (long-assessment 1) - at the end of the intervention (12 months) (long-assessment 13)	100 100	80 85	100 100	100 100	-
Marital status, n (%) - Single - Married/partner - Divorced - Widowed	11 (27.5) 18 (45) 9 (22.5) 2 (5)	7 (50) 3 (21.4) 4 (28.6) -	3 (23.1) 5 (38.5) 3 (23.1) 2 (15.4)	1 (7.7) 10 (76.9) 2 (15.4) -	1 (5.6) 15 (83.3) 2 (11.1) -
Children, n (%) - Yes - No	22 (55) 18 (45)	8 (57.1) 6 (42.9)	8 (61.5) 5 (38.5)	6 (46.2) 7 (53.8)	14 (77.8) 4 (22.2)
Native language, n (%) - German - Other - N.a.	37 (92.5) 3 (7.5) -	13 (92.9) 1 (7.1) -	13 (100) - -	11 (84.6) 2 (15.4) -	16 (88.9) 1 (5.6) 1 (5.6)
Patient advance directives, n - Yes - No	16 (40) 24 (60)	5 (35.7) 9 (64.3)	4 (30.8) 9 (69.2)	7 (53.8) 6 (46.2)	-
Patient decree - Yes - No	12 28	4 (28.6) 10 (71.4)	2 (15.4) 11 (84.6)	6 (46.2) 7 (53.8)	-

SD = Standard Deviation, EDSS = Expanded Disability Status Scale, GdB = Grad der Behinderung/degree of disability

In 15 cases, PwsMS lived alone, in 20 cases with a partner, in nine cases with their child(ren), in one case the PwsMS lived with two partners (one of whom participated as a caregiver), in one case the caregiver was the parent of the PwsMS and they lived together, and in one case the PwsMS and caregiver were separated but shared the accommodation.

### Numbers analyzed

The mITT population of the intervention group consists of 40 PwsMS, 18 of whom had a caregiver. All participants randomized to the intervention group were also evaluable in accordance with the mITT principle. Consequently, all data collected in the intervention group were included in the analysis.

### Characteristics of the care and case managements personal contacts

The average duration of the short-assessments was 63 minutes (range: 18–160 minutes, SD: 35 minutes), while the average duration of the long-assessments was 91 minutes (range: 26–230 minutes, SD: 49 minutes).

The average time of the short-assessments was reduced from 106 minutes (range: 30–300 minutes, SD: 70 minutes) (SA2) to 49 minutes (range: 5–120 minutes, SD: 25 minutes) (SA12). The average time for the long-assessments was reduced from 174 minutes (range: 70–420 minutes, SD: 96 minutes) (LA1) to 73 minutes (range: 20–120 minutes, SD: 21 minutes) (LA13).

### Care plan and goal achievement

In total 1025 goals and 1025 related actions were documented due to an unmet need in the care plan. The reason why the number of objectives and actions is exactly the same is based on the study specific structure of the CCM Manual [25]: This stipulated that every action must be linked to a goal.

**Fig. 3:**
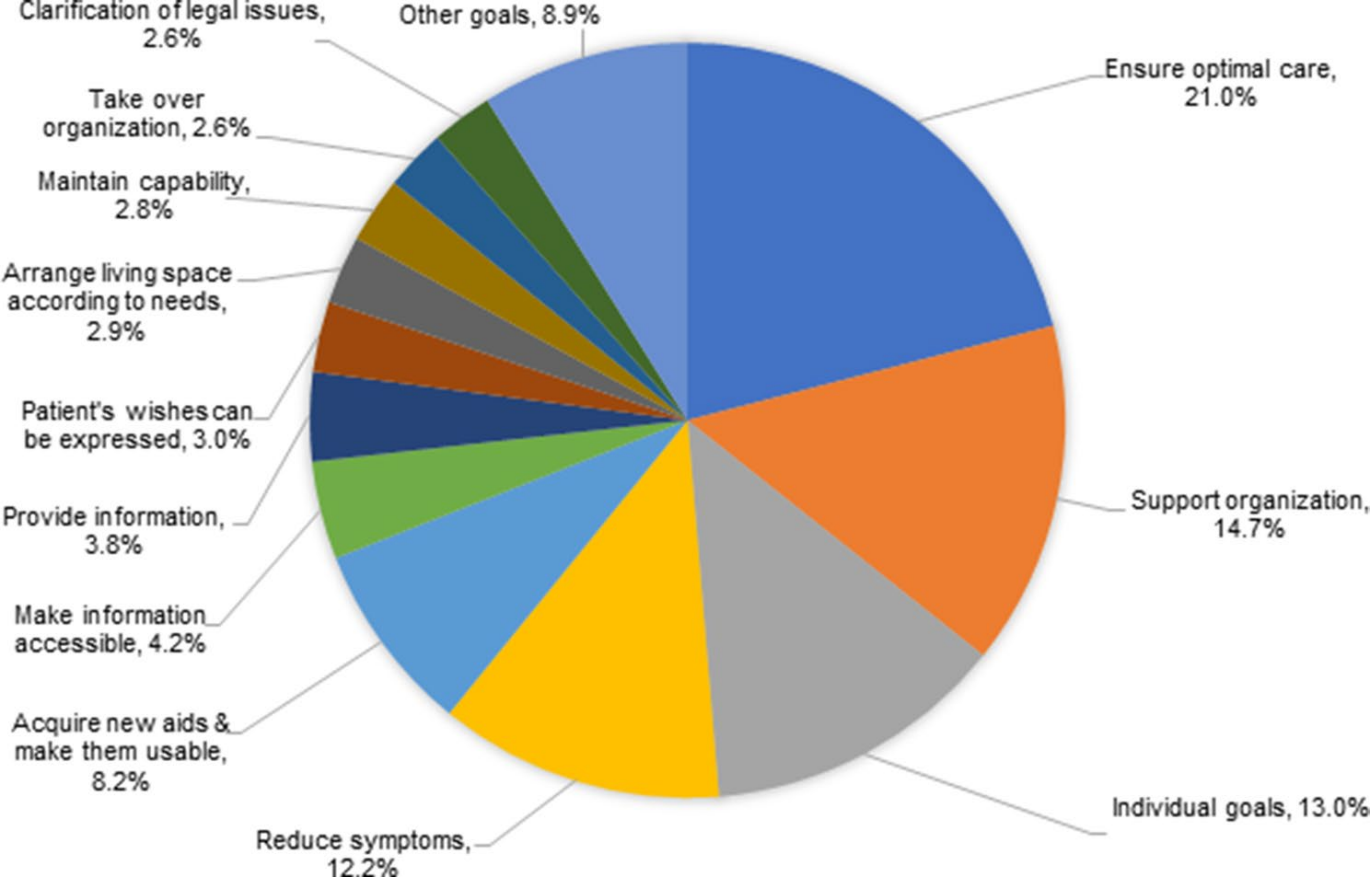
Domains of the specified goals of study participants

The four most frequently documented *goal* categories encompassing 61% of all defined goals were: ensuring optimal care (*n* = 215, 21%), supporting organization (*n* = 151, 14.7%), individual goals (related to relief through conversation, support in decision making about medical issues/therapies, social issues, and practical help) (*n* = 133, 13%), and reducing symptoms (*n* = 125, 12.2%) (see Fig. 3).

**Fig. 4:**
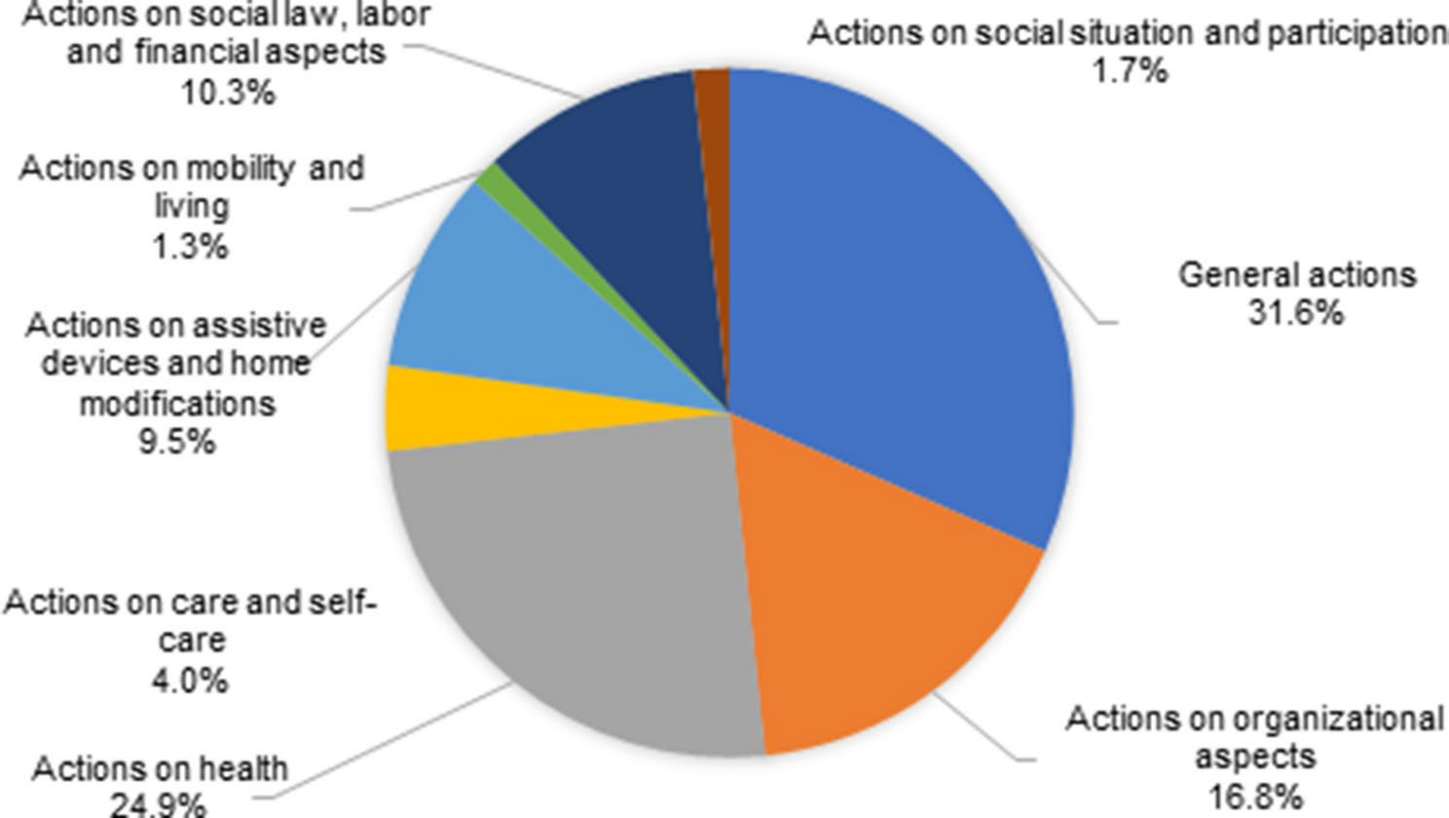
Most frequent domains of actions in the care plan to achieve the defined goals

Among the implemented 1025 actions to achieve the set goals, those of a general (e.g. obtaining and providing information, advice) (31.6%), health-related (24.9%), and organizational nature (16.8%) were the most prevalent. The frequency of the thematic domains of actions are presented in Fig. 4.

At the end of the CCM process the majority of goals (77.2%) were achieved, while 13.4% could not be achieved. The most frequently cited reasons for the failure of goal achievement were a lack of suitable cooperation partners (accessible facilities, capacity) or the goal was not required by PwsMS and caregivers any longer.

### Impact of care and case management on dimensions of life

Table 4 illustrates the significant changes observed in the various dimensions of life during the CCM process from initial to last assessment of CCM intervention. Notably, there were significant changes observed in the domains: physical health, self-sufficiency and social situation and participation. In alignment with the chronic progression of the disease, resources demonstrated scarcely significant improvement. The aspect of physiotherapy showed a significant worsening of problems throughout the intervention. However, with the support of the CCM, problems and particularly resulting unmet needs exhibited noteworthy significant improvement.

**Table 4:** Comparison of problems, resources and needs: initial assessment (LA1) vs. last assessment (LA13), *p*-value is calculated using the sign test (α < 0.05); light grey meaning improvement, dark grey meaning deteriorations

Dimension	Area	Problems	Resources	Needs	p
**Physical health**	MS-specific health			x	0.039
	General health status			x	0.021
**Self-sufficiency**	Self-care in the household and organization			x	0.039
	Assistance with housing and living conditions			x	0.039
	Housing			x	0.039
	Medical nursing, therapeutic care	x			0.008
				x	0.002
	General practicioner		x		0.021
	Other specialist care	x			0.039
				x	0.039
	Outpatient care service			x	0.016
	Physiotherapy	x			<0.001
	Psychotherapy			x	0.039
**Social situation and participation**	Involvement of family and friends	x			0.006
	Pursuing hobbies and interests			x	0.002
	legal issues			x	0.002

### Development of unmet needs during the CCM process

The following section presents a comparison of the resulting PwsMSs’ unmet needs in 30 sub-domains of life as measured at the beginning of the CCM process (initial assessment = long-assessment 1) with those at the end of the CCM process (final assessment = long assessment 13). The green bars and numbers in the green boxes in the tables and figures represent improvements, those in red represent deteriorations, and those with no or no specific changes of unmet needs in that area are listed in grey. The most commonly reported unmet needs were ‘MS specific’ (*n* = 33, 86.8%), ‘mental health’ (*n* = 27, 71.1%) and the least commonly reported unmet needs were ‘coordination of appointments’, ‘daily structure’ and ‘speech therapy’ *n* = 2 each (5.9%-10%). Overall, there were 184 cases of improvement and 47 cases of deterioration in the 30 sub-domains for the 38 progressively diseased PwsMS between the initial and final assessments; on average 6.3 improvements and 1.6 deteriorations were recorded per sub-domain.

#### Physical health

**Fig. 5:**
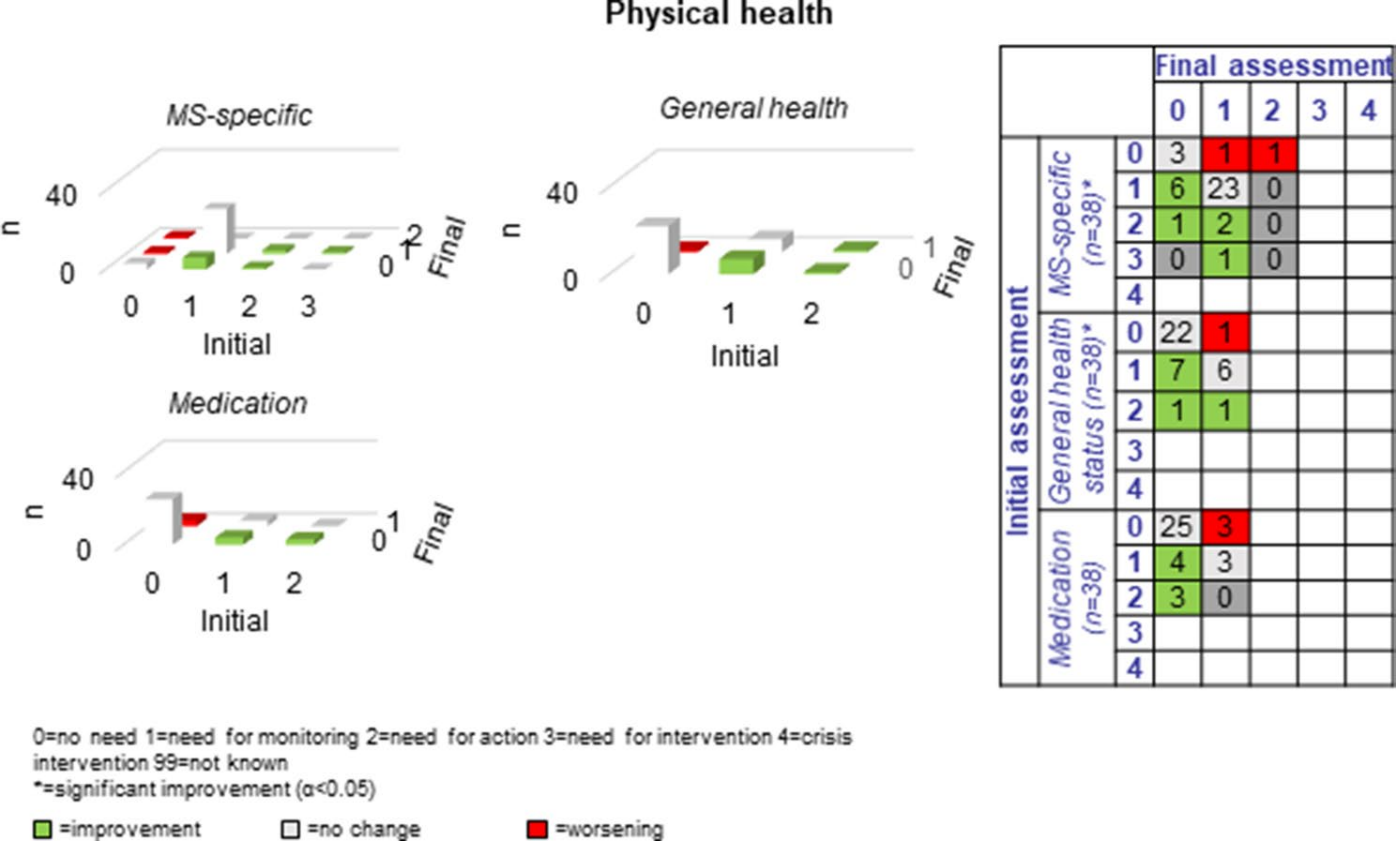
Development of physical health during the CCM intervention

At the beginning of the CCM process the vast majority (*n* = 33, 86.8%) of PwsMS demonstrated unmet needs with respect to “MS-specific physical health”; ten (30.3%) improved. Fifteen (39.5%) exhibited unmet needs in the dimension “general health”; nine of them (60%) improved. Ten (26.3%) exhibited unmet needs in the dimension “medication”; seven of them (70%) improved (see Fig. 5).

#### Mental health

**Fig. 6:**
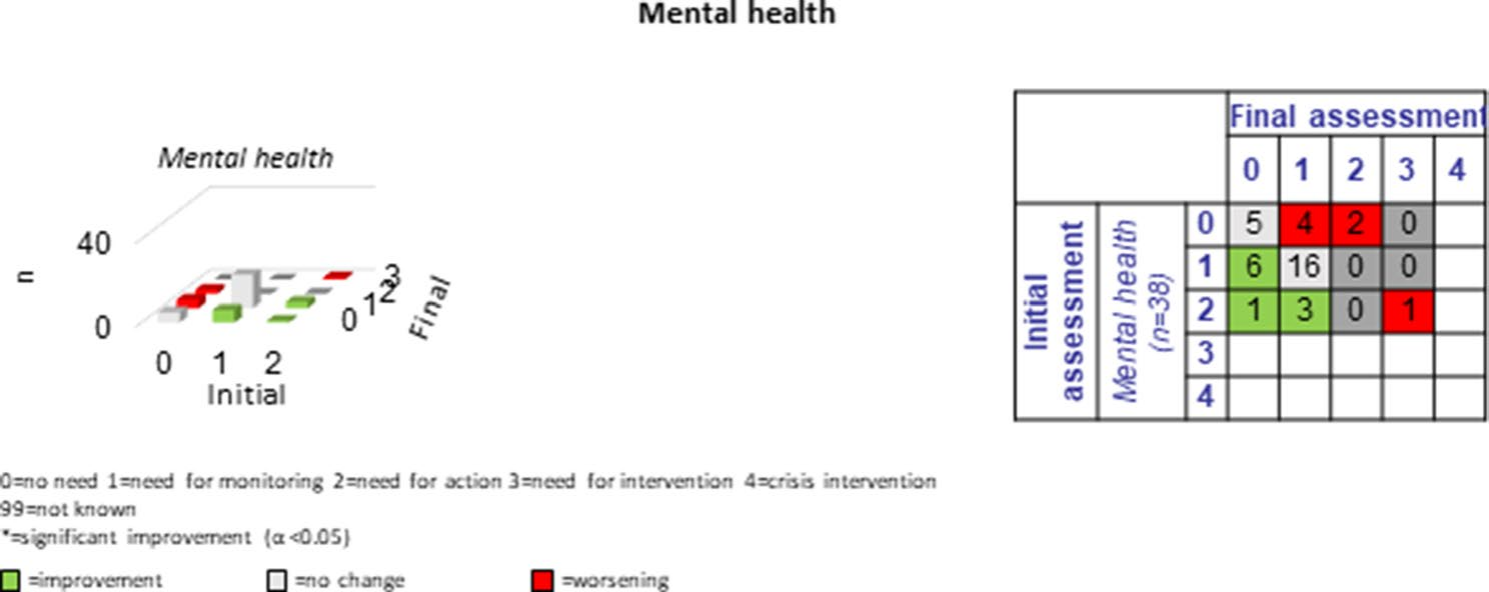
Development of mental health during the CCM intervention

Of 27 (71.1%) showing an unmet need initially, ten (37%) improved; however, seven (25.9%) worsened (see Fig. 6).

#### Autonomy:

**Fig. 7:**
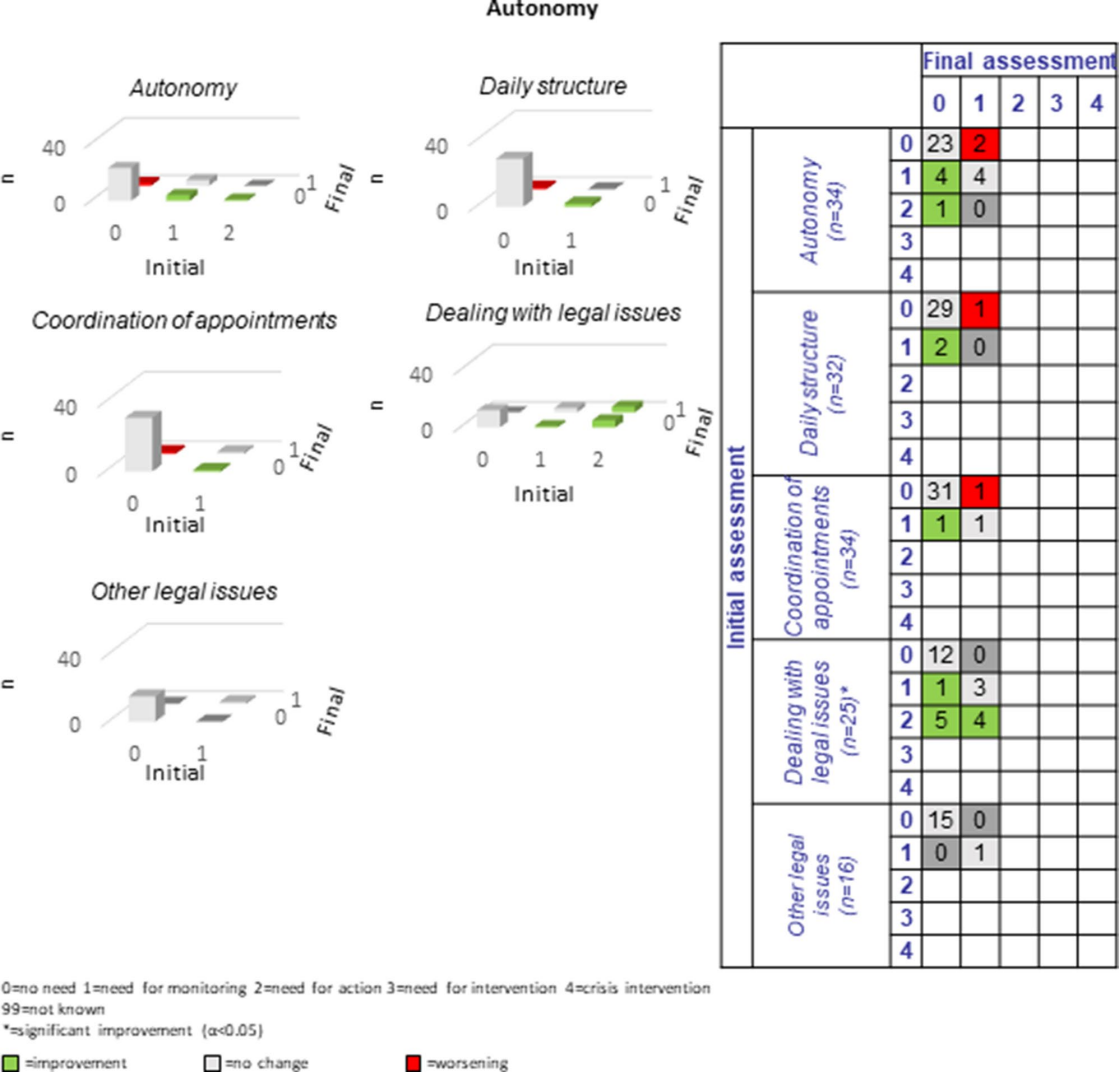
Development of autonomy during the CCM intervention

With regard to *autonomy* in detail one to 13 PwsMS (5.9%-52%) showed unmet needs in the presented sub-domains. The majority improved which was especially true for the significant sub-domain “dealing with legal issues”: Ten of 13 PwsMS improved, meaning 76.9% (see Fig. 7), showing that this was a crucial factor so far paid sparse attention to.

#### Self-sufficiency:

**Fig. 8:**
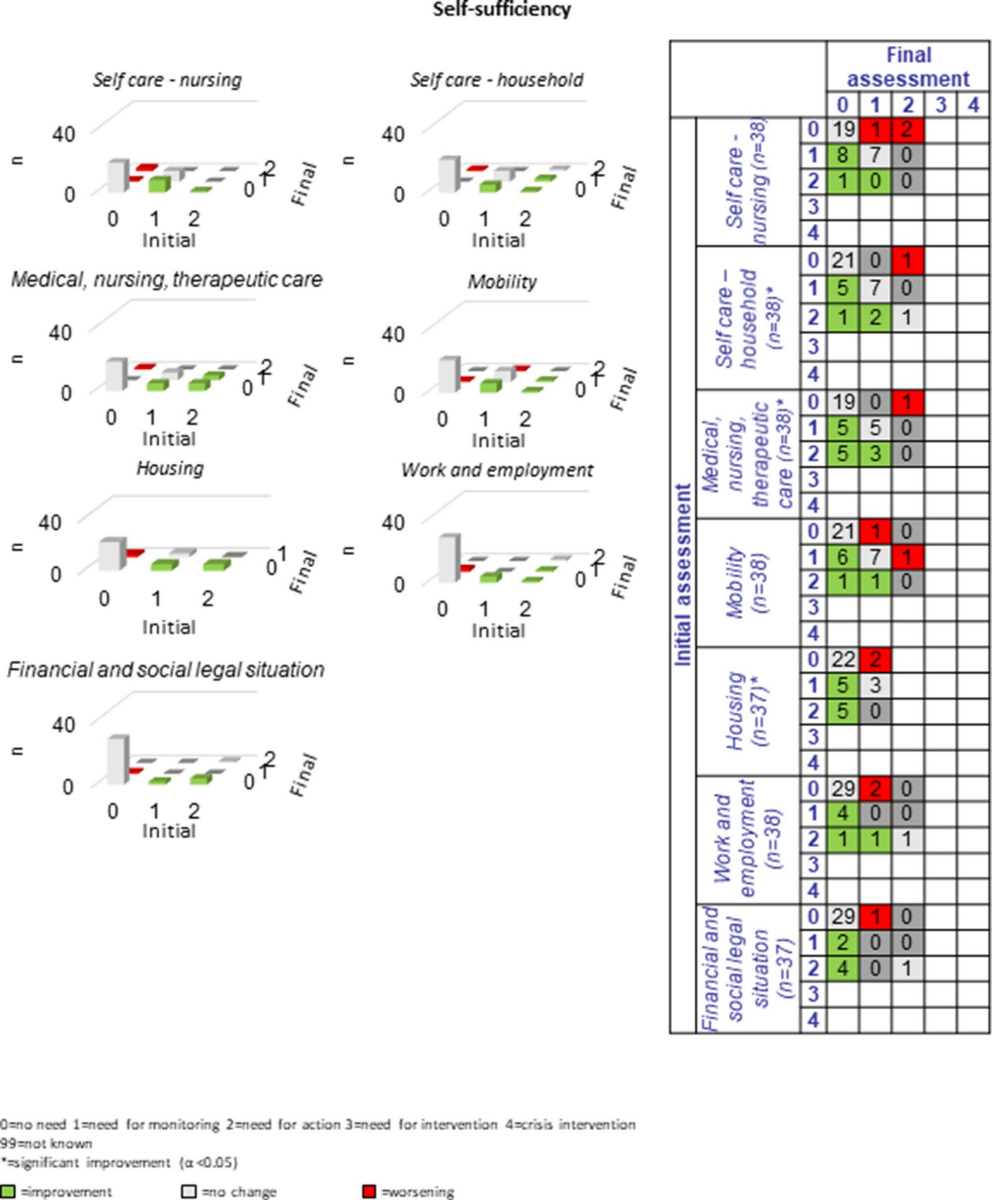
Development of self-sufficiency during the CCM intervention

Restrictions in *self-sufficiency* were an aspect of special relevance: Within the seven sub-domains up to 18 (47.4%) exhibited unmet needs and nine to 15 PwsMS (50%-85.7%) of these demonstrated improvements in the respective sub-domains at the end of the CCM process (see Fig. 8). The domain *self-sufficiency* was the one with the most significant changes, highlighting CCM’s expertise in increasing independence, where this is possible in the context of a chronic illness.

#### Medical, nursing, and therapeutic care

**Fig. 9:**
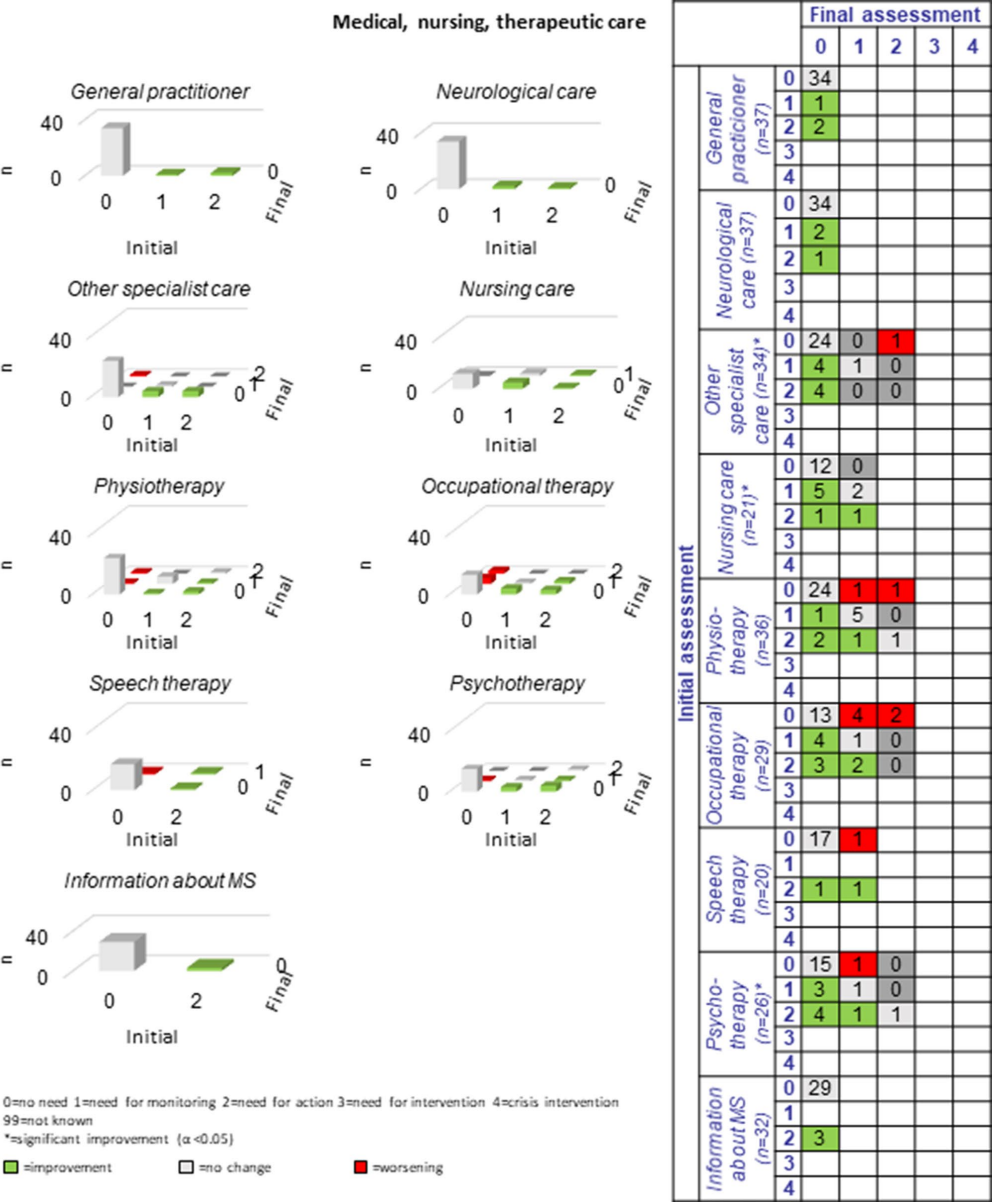
Development of medical, nursing, therapeutic care during the CCM intervention

With regard to *medical, nursing, and therapeutic care* in greater detail two to ten (8.1%-42.9%) PwsMS reported unmet needs at the beginning of the CCM process; leading sub-domains were “occupational therapy”, “physiotherapy” and “psychotherapy”, each of them with ten PwsMS showing unmet needs (27.8%-38.5%). In most cases unmet needs in the diverse sub-domains could be met with help of the CCM which is especially with respect to “psychotherapy” treatment options really worth mentioning as disabled access is normally rare. Most worsening processes could be observed in the sub-domains “occupational therapy” (*n* = 6) and “physiotherapy” (*n* = 2) (see Fig. 9). Although the unmet needs of most of the PwsMS who identified them as a problem at the beginning of the CCM intervention could be met, other PwsMS developed new unmet “occupational” and “physiotherapy” needs during the CCM period probably due to their progressive disease, which could not be met in time by the end of the CCM process.

#### Social situation and participation:

**Fig. 10:**
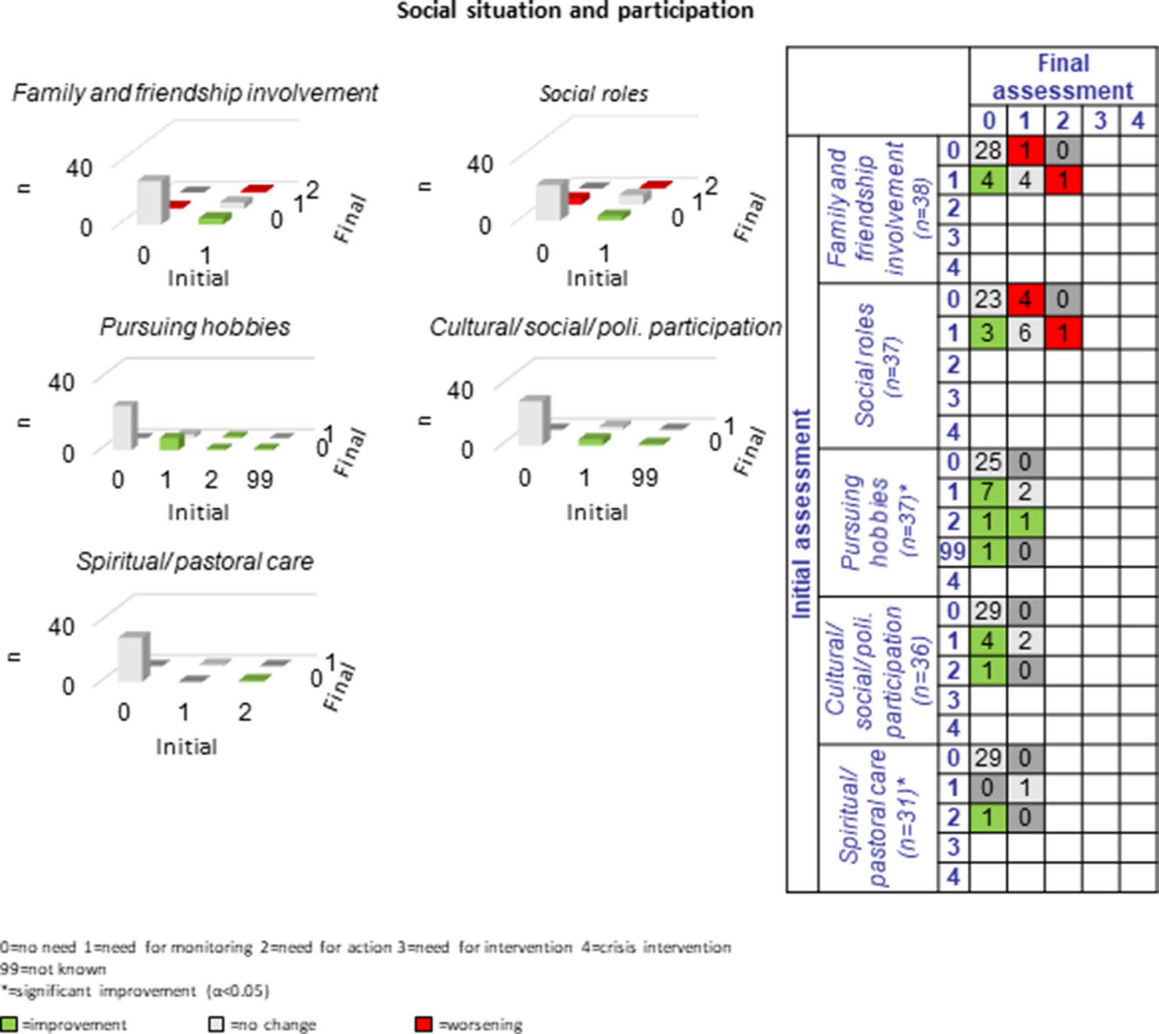
Development of social situation and participation during the CCM intervention

The need for improvement in the five sub-domains of *social situation and participation* was between two and twelve (6.5%-32.4%). Improvement was most often achieved in the sub-domain “pursuing hobbies” (*n* = 10, 83.3%). Other sub-areas also improved, however, in fewer cases (*n* = 1–5, 30%-83.3%). Worsening processes could be observed in the sub-domain “family and friendship involvement” (*n* = 2) and “social roles” (*n* = 5) (see Fig. 10). Even longer-term CCM processes as offered here are presumably necessary, as aspects such as friendships, lifestyle or organization of social roles are complex individual characteristics that have developed over the long-term decades.

#### Caregivers

The 18 caregivers participating in the study showed considerably variable assessment participation: Eight caregivers participated in 11–13 CCM assessments, six caregivers participated in 6–9 assessments, and four caregivers participated in 1–5 assessments.

**Fig. 11:**
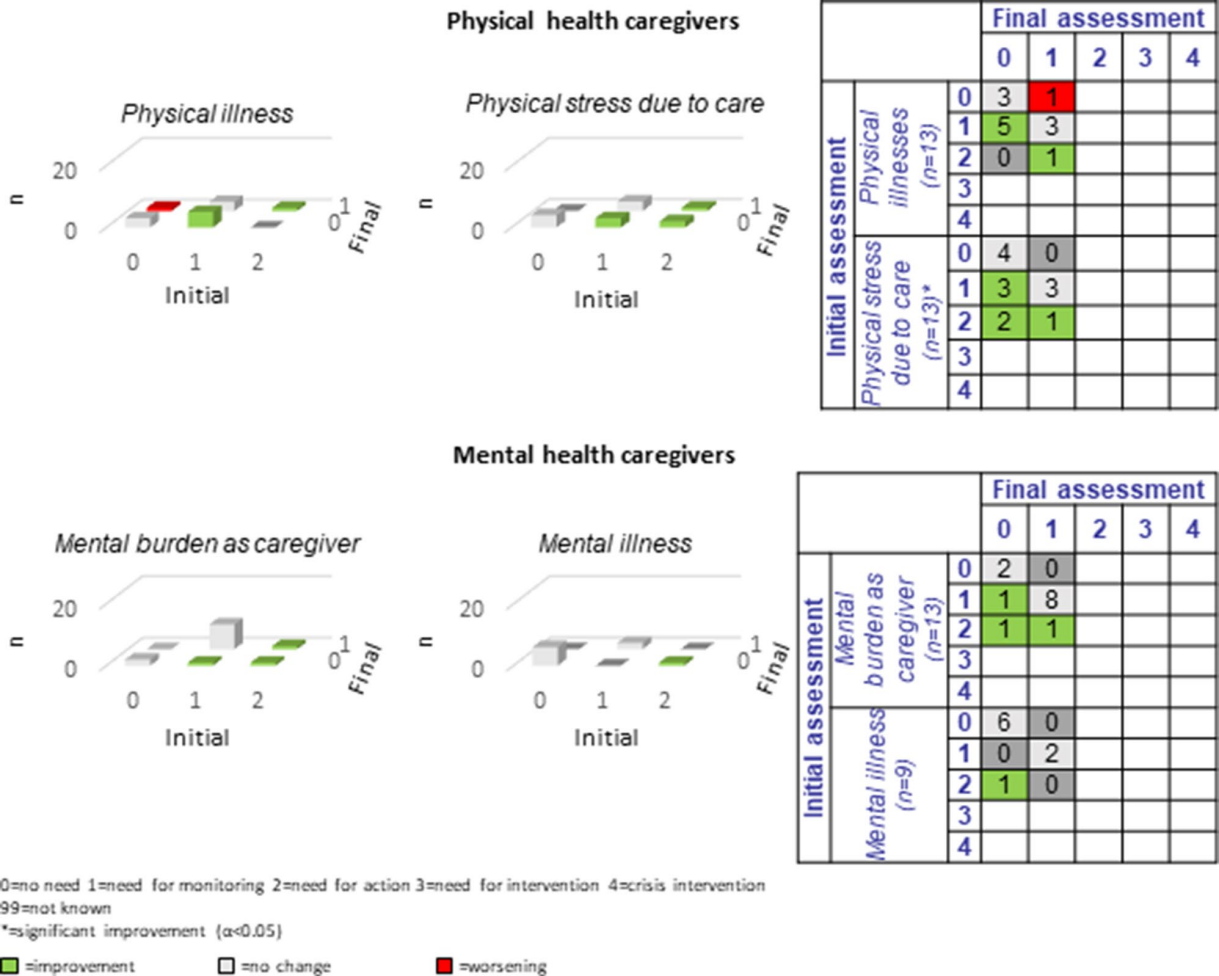
Development of caregivers’ physical and mental health during the CCM intervention

**Fig. 12:**
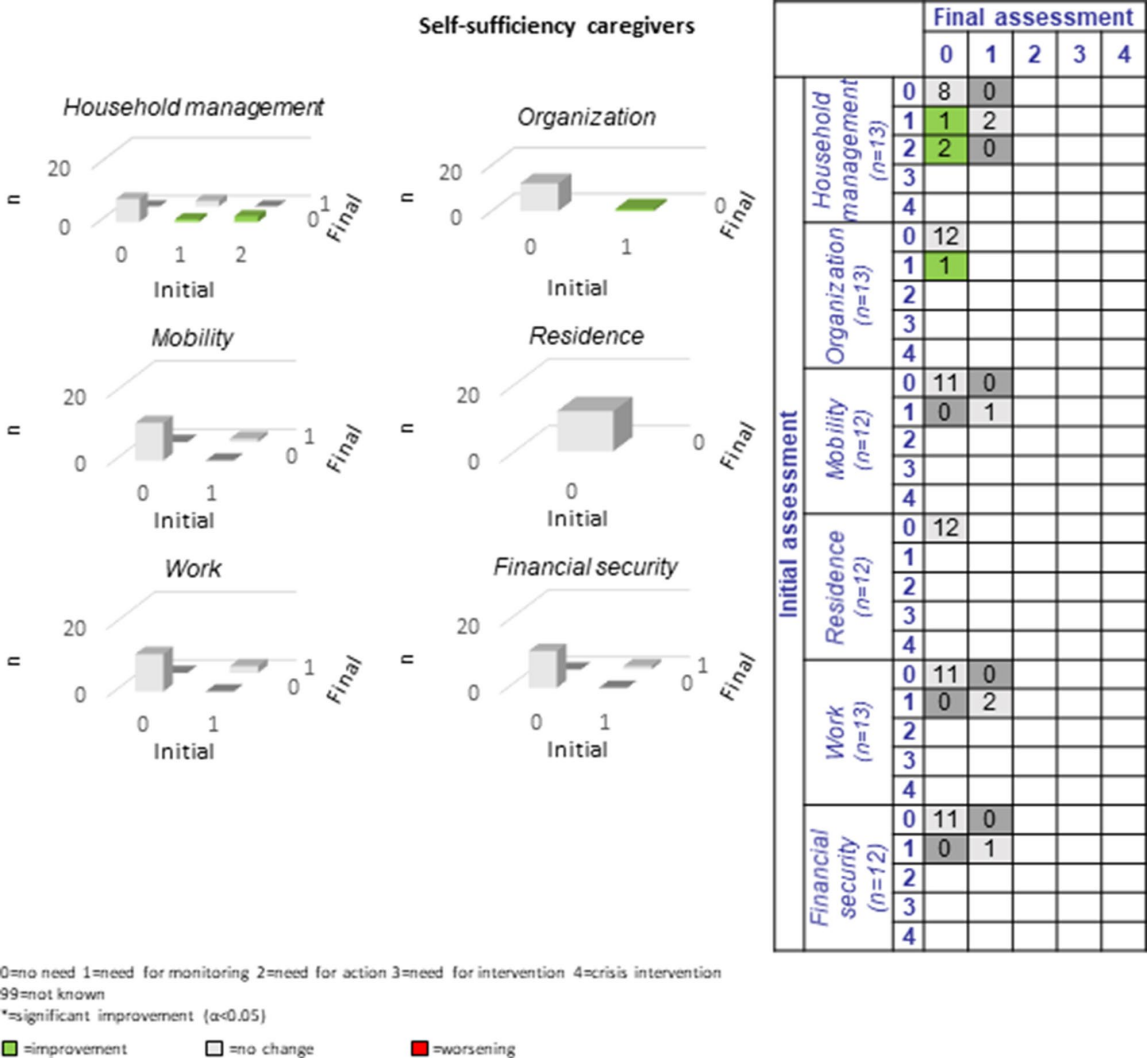
Development of caregivers’ self-sufficiency during the CCM intervention

**Fig. 13:**
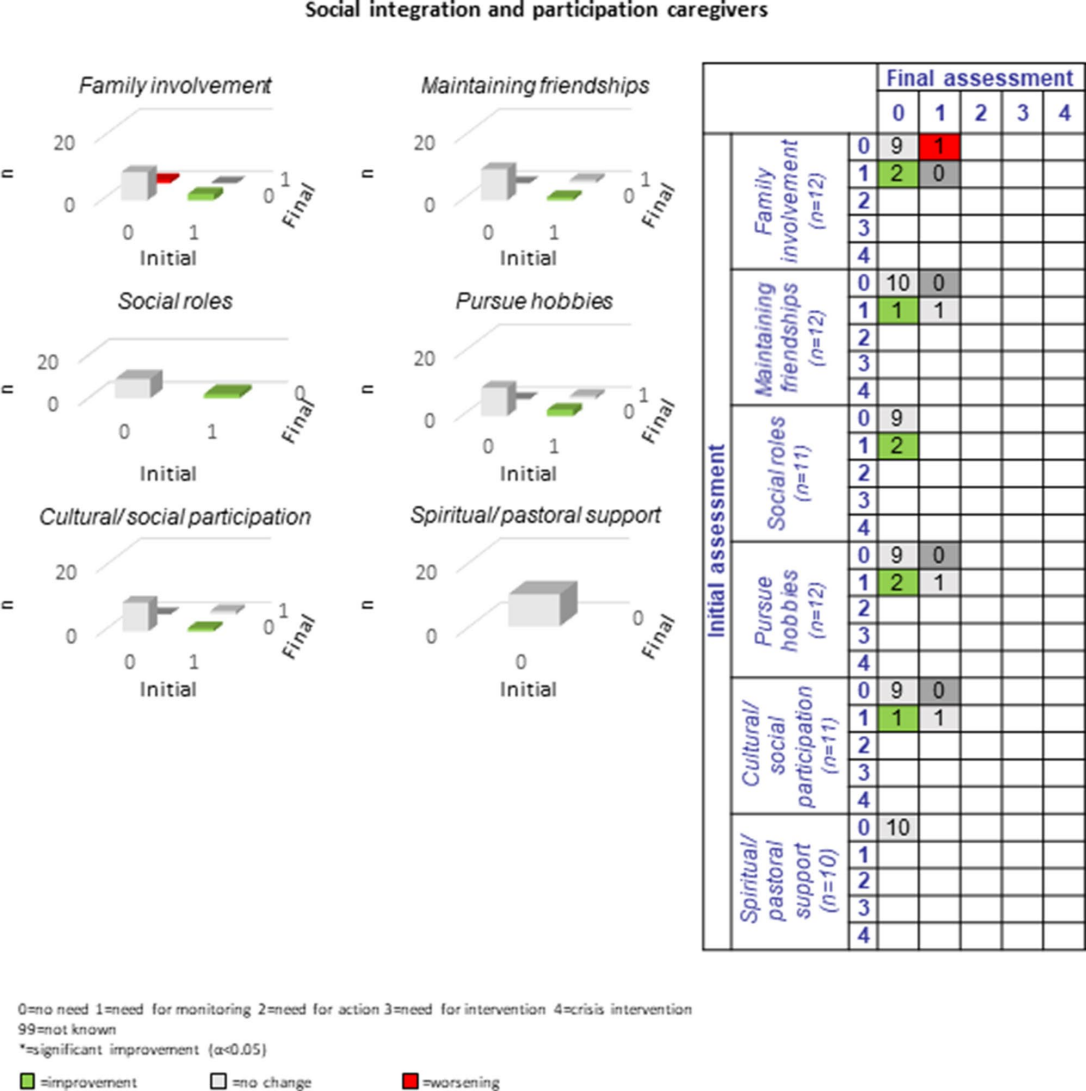
Development of caregivers’ social integration and participation

Across the total of 16 caregiver domains, an average of 1.8 improvements and 0.1 deteriorations were recorded per sub-domain (see Figs. 11, 12 and 13). Specifically, only two cases of deterioration were documented (compared to 28 improvements), one in relation to *physical health*, sub-domain “physical illness”, and one in relation to *social integration and participation*, sub-domain “family involvement”. Participating caregivers reported most unmet needs in the categories of *physical and mental health* (*n* = 9–11; 69.2%-84.6%) and no unmet needs in the sub-domains of “residence” and “spiritual care”.

#### Care management portfolio

**Fig. 14:**
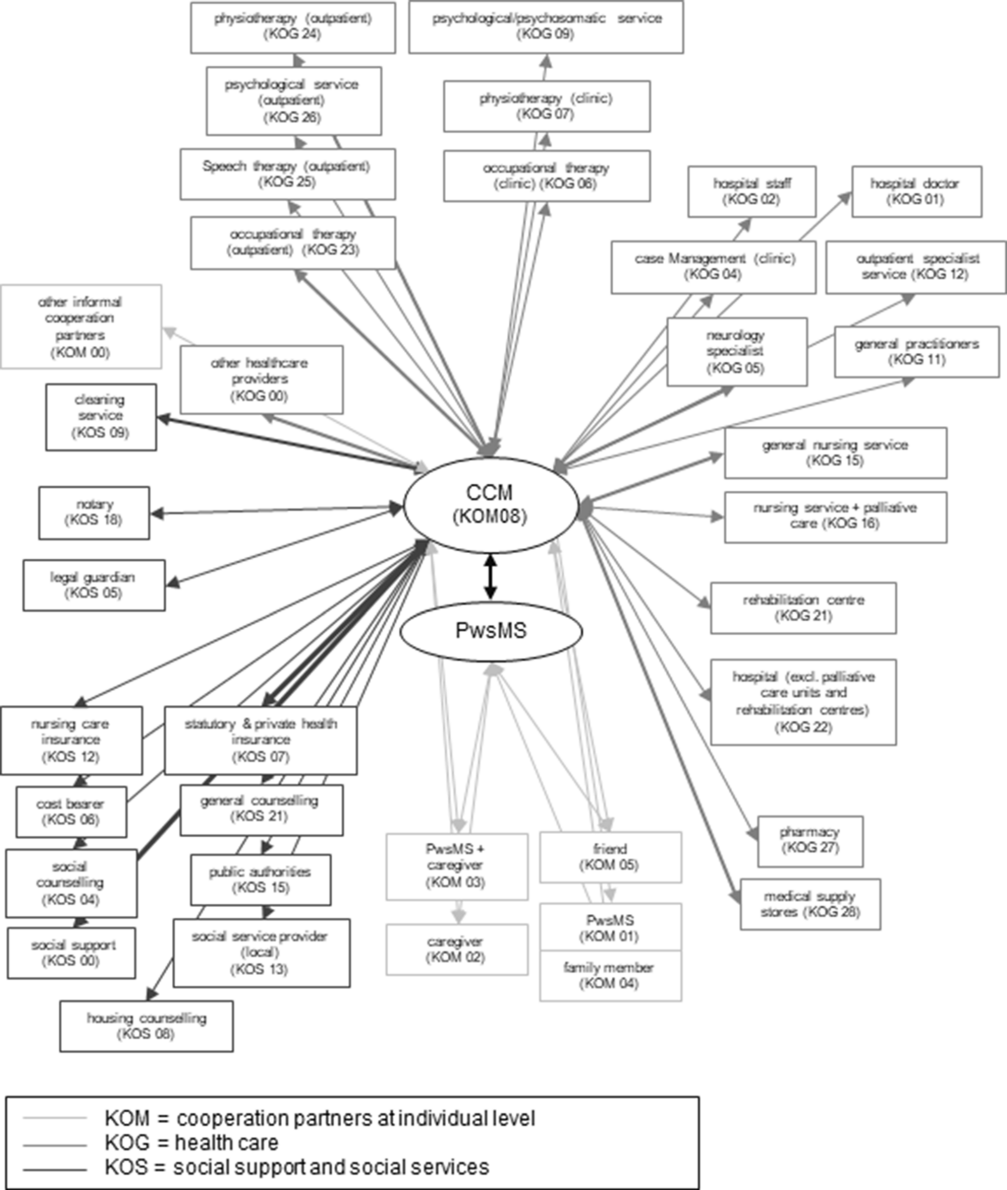
Network of care management. The thickness of the arrows indicates the quantity of contacts (thin line - few contacts, thick line - many contacts)

During the CCM intervention, the care and case managers listed cooperation partners (*n* = 1025) with whom contact was established in order to implement actions to achieve study participants’ goals. In total, 39 different disciplines were listed (see Table 5). In most cases (*n* = 616, 60.1%) the care manager “cooperated” with herself meaning she solved the problem by own actions or the PwsMS could manage by him/herself encouraged by the case manager (*n* = 54, 5.3%). The six further major partners were: medical supply stores (*n* = 40, 3.9%), outpatient physiotherapists (*n* = 32, 3.1%), neurologists (*n* = 31, 3%), and specialists (*n* = 29, 2.8%). Table 5 shows all disciplines of cooperation partners listed throughout the CCM intervention and Fig. 14 shows which cooperation partner cooperated with whom underlining the complexity of the overriding care management.

**Table 5:** List of cooperation partners mentioned in the care plans

Cooperation partner Care plan		
	Amount	%
Study CCM (meaning the study CCM did by herself without a further cooperation partner)	616	60.1
Patient (meaning the study participants did encouraged by the case manager)	54	5.3
Medical supply store	40	3.9
Physiotherapy (outpatient)	32	3.1
Specialist (neurology)	31	3.0
Outpatient Specialist doctor	29	2.8
Social support Other	26	2.5
Occupational therapy (outpatient)	20	2.0
Health care: Other	19	1.9
Psychological/psychosomatic service (outpatient)	18	1.8
Cleaning service	17	1.7
General practitioner	13	1.3
Statutory health insurance/private health insurance	12	1.2
Family member	11	1.1
Nursing service (general)	11	1.1
Caregiver	11	1.1
Cost bearer	7	0.7
Clinical doctor	7	0.7
Legal guardian	6	0.6
Speech therapist (outpatient)	5	0.5
Rehabilitation center (outpatient/inpatient)	4	0.4
Hospital (palliative care & rehabilitation unit excluded)	4	0.4
Social counseling center	4	0.4
Patient and caregiver together	4	0.4
Other informal cooperation partner	3	0.3
Housing counseling	3	0.3
Social benefit providers (local)	3	0.3
Public offices	3	0.3
Nursing care insurance	2	0.2
Clinic nursing staff	1	0.1
Case management (clinic)	1	0.1
Occupational therapy (clinic)	1	0.1
Physiotherapy (clinic)	1	0.1
Psychological/psychosomatic service (clinic)	1	0.1
Nursing service (with general palliative care)	1	0.1
Pharmacy	1	0.1
Friend	1	0.1
Notary	1	0.1
Advice center	1	0.1
Total	1025	100.0

A cross-table analysis revealed that the main goal which the CCM worked on was to ensure optimal care (18.7% of all goals carried out by the CCM). When PwsMS worked on a goal, they aimed most frequently to procure and install new assistive devices (14.8%) and getting in contact with medical supply stores (45%). Neurologists were most frequently aiming to ensure optimal care (32.3%) and to reduce symptoms (32.3%). Outpatient physiotherapists were most often occupied with reducing symptoms (34.4%). The most frequent goal of other outpatient specialists (urologists, dermatologists, gynecologists, dentists, and ophthalmologists, as well as cardiologists, psychiatrists, pain therapists, rheumatologists, orthopedists, and internists) was to ensure optimal care (37.9%), meaning to optimize care individually for the PwsMS (e.g. optimized therapy like medication, treatment according to the individual needs like providing information or support in organizing everyday life).

## Discussion

PwsMS benefit from the long-term cross-sectoral CCM in all dimensions of life.

First and foremost, physical and mental health and care and organizational aspects were addressed and improved through the CCM process. Unmet needs in these areas played the most important role for both PwsMS and caregivers. This is despite the fact that the German health and social care system is characterized by a well-developed structure [42]. One reason for this finding is that the system is fragmented and PwsMS and their caregivers are overwhelmed by the unmet need to find their way around. This is especially true in the case of people with severe illnesses as studied here, as mental and physical resources decline with disease progression and caregivers’ burden increases [43–45]. Here, the CCM becomes relevant, as an objective counterpart to the patient’s journey through the disease. Over time, patients’ resources are enhanced by the CCM, thereby increasing their self-sufficiency and enabling them to address an increasing number of tasks independently. This means their health literacy [46] improves, enabling them to manage their illness better and to make self-directed decisions in relation to their illness and associated areas of life. This, enhances participation and biographical reorganization. The increase in patients’ self-sufficiency in various sub-domains belonged to most evident changes through the CCM process. Unmet needs, goal setting, and goal achievement in the domains and sub-domains of autonomy, social situation and participation were less frequently mentioned in this study than in relation to the health and social care situation. However, they were present and showed significant or tentative improvement. It is to be expected that once the basic health and social care problems have been resolved, these other aspects will become more prominent and require more action. This suggests that the CCM process should not be limited to a fixed time period (in this case 12 months limited through study duration) [47], but should continue throughout the patient’s journey as their unmet needs change [48, 49]. This is a recommendation also given by others dealing with case management or so called “patient guides” in the health care system [50]. Through a patient journey, intensity of caring and time spent on by the CCM will change over time, with peak at the beginning and then on an ad-hoc basis, depending on unmet needs during the course as reflected in our documented decrease in time for CCM assessment contacts. This development is possible by the familiarity that grows between the CCM and the patient and caregiver, if applicable. The CCM can facilitate improved access to medical treatment and daily living support for serious chronic illness, which has been reported to slow disease progression, improve quality of life and help patients regain their social roles [22, 51–54]. In terms of what CCM makes possible for patients and caregivers, but also in terms of time saved by healthcare specialists, the effort involved in long-term, continuous CCM appears to be manageable as we needed 1.2 to 1.3 full time working care and case managers to care for 38 patients in our study.

A goal achievement of nearly 80% clearly speaks in favor of the CCM intervention, this especially as the most present reasons for not achievement were lacks in the health and social care system. This means that certain cooperation partners were simply not sufficiently implementable, one example being (home based) occupational- or physiotherapy. Psychotherapy, however, could with some effort be adequately installed. Although mental health problems have improved for a considerable number of our patients studied here as a result of the CCM, the need of psychotherapy will remain high, as new mental health problems have arisen, which is not surprising given that an estimated 30.5% of people with MS have a prevalence for depression [55], and the prevalence for mental health (comorbidity) issues is high [55–57]. 22% of severely affected MS patients report suicidal thoughts [58] and a suicide rate two times higher as in general population [21, 59–62]. The CCM could play a crucial role here, not only for careful, confidential risk assessment with initial relief, but also for (risk) intervention by involving general practitioners (GPs) or medical specialists and helping to find a treatment. In terms of suicide prevention, the CCM points in exactly this direction, as our results are encouraging in terms of increased self-sufficiency or increased pursuit of one’s own interests and hobbies. Both promote participation and regaining life roles. These effects are components of suicide prevention [21].

The variety and number of activities carried out by the CCM reflects the high level of complexity of the unmet needs arising from resource- and problem-analysis of PwsMS and their caregivers, which change with individual disease trajectories [4–8]. The CCM herein is flexible, adaptive, and able to address the variety of individual goals through continuous monitoring and adjustments. During the study it became evident that individual goals and actions were even more specific than previously defined in the CCM manual (36 predefined goals, 125 predefined actions); this result helps us in adapting the assessment for future studies and implementation.

Collaboration partners were multidisciplinary and varied according to the patient’s symptoms and disability status. This enormous network across insurance systems that has to be activated for these patients in order to provide them with adequate care is overwhelming for the patients themselves. However, the burden of overall care management should not be underestimated for the CCM: In accordance with the DGCC we estimate that almost 30% of their working time is spent on overall care management. The rest of the time is needed for the individual case management. CCM of PwsMS requires a multidisciplinary approach. In addition to certified CCM training, CCMs would benefit from MS and complex disease-specific expertise and interaction with healthcare specialists. This could be achieved, for example, by locating them either in neurological facilities or clinics specializing in MS, or in larger community GP practices, and by additional inter- and multi-professional regional networking bringing together healthcare specialists from different disciplines, a potential of the CCM structure. This approach could also help healthcare specialists to recognize the lack of collaborative partners and the general lack of care for people with MS. Generous care management would then mean creating new care structures on the basis of existing structures in order to fill the gaps in care.

### Strengths and limitations

In this study, we were able to recruit exactly the PwsMS without delay – indicating an unmet need - and get them interested in the study. In many MS studies, these patients are not recruited because they do not fit to study protocols or they are simply not found in the healthcare system because they cannot reach the necessary healthcare structures any longer due to e.g. side effects of medications like fatigue, immobility, and facilities not suitable for disabled people or accessibility of buildings. This is one of the strengths of our study. We recruited in rural and urban districts of Cologne (4.54 million inhabitants) under the direction of the Departments of Palliative Medicine and Neurology at Cologne University Hospital, through outpatient and inpatient neurological institutions, the German Multiple Sclerosis Society (DMSG) registry, and the local DMSG group in Cologne. The latter, in particular, helped us to acquire peer group members. However, the results, some of which are already significant or at least point in the right direction, indicate the importance of this form of care and encourage follow-up projects aimed at implementing this care structure in the healthcare system. Notabene, due to the lack of adjustment for multiple testing our results should be interpreted with caution and viewed as preliminary. These follow-up projects can be designed precisely on the basis of this exploratory study. The CCM intervention appeared to be less beneficial for caregivers than for patients. However, this was not the primary focus of the trial. We know from caring for people with complex serious illnesses such as PwsMS that their caregivers are enormously important in the so-called ‘unit of care’. Future projects should pay particular attention how to address the caregivers of these patients. The results of the study cannot be extrapolated to the whole of Germany, but it is possible to extrapolate to the extent that the administrative district of Cologne (a region of 7365 km^2^ far beyond Cologne) is a geographical area that and includes both urban and rural districts, and that the patient study population exactly reflects the target study population with unmet needs of PwsMS, as well as the type of intervention being internationally comparable.

## Conclusion

The CCM intervention was beneficial for those affected in a multitude of ways, with approximately 80% of the individual set goals being met. The successful work of the CCM on the individual and overarching levels reflects the complexity of care necessity and unmet needs of this patient population. Enhanced health literacy empowers patients to manage arising future healthcare problems and moreover allows them to strengthen their identity apart from their disease due to released resources. A gain in self-sufficiency, resilience and participation may work in a suicide preventive manner. This hypothesis has to be tested in a further study. This exploratory clinical trial managed to address the severely affected MS patients, a study population so far underrepresented in clinical trials. The results of the study will help to design future in-depth studies with the long-term goal of including this type of care in standard care. MS is a prime example of a long-term neurological condition. Due to demographic changes, the significance of these diseases will increase in the future. Future studies must evaluate whether a CCM intervention could be beneficial for other long-term conditions besides MS.

## Electronic supplementary material

Below is the link to the electronic supplementary material.

Supplementary material 1

## Data Availability

Generated and/or analyzed datasets of participants are available from the corresponding author on reasonable request to protect participants.

## Abbreviations

CCM: Care and case management
CM: Case Management
CNS: Central nervous system
COCOS-MS: Communication, Coordination and security for people with multiple sclerosis
DGCC: Deutsche Gesellschaft für Care und Case Management
DMSG: Deutsche Multiple Sklerose Gesellschaft/German Society for Multiple Sclerosis
DRKS: German register for clinical studies
EDSS: Extended disability status scale
GdB: Grad der Behinderung/degree of disability
GP: General practitioner
LA: Long assessment
mITT: Modified Intention-to-Treat
QOL: Quality of life
MS: Multiple sclerosis
PwsMS: Persons with severe multiple sclerosis
SA: Short assessment
SD: Standard Deviation

## Declarations

### Protocol

The study protocol is published [21].
