# Supplementary material for: Long-term cross sectoral care and case management for people with severe multiple sclerosis and their caregivers – impact on dimensions of life
Source: BMC Health Serv Res. 2026 Mar 14;26:488. doi: 10.1186/s12913-026-14177-y (PMC13064002; doi:10.1186/s12913-026-14177-y)
Supplement: Supplementary file 1 — Supplementary material 1 [file 12913_2026_14177_MOESM1_ESM.docx]

**Predefined goals and actions referring to the CCM manual**

A. Goals

| Code | Goals |
| --- | --- |
| ZIE 00 | Individual goal |
| ZIE 01 | Maintain weight |
| ZIE 02 | Gain weight |
| ZIE 03 | Lose weight |
| ZIE 04 | Regulated consumption |
| ZIE 05 | Abstinence |
| ZIE 06 | Understand symptoms |
| ZIE 07 | Reduce symptoms |
| ZIE 08 | Symptom free |
| ZIE 09 | Ensure optimal care |
| ZIE 10 | Enable access |
| ZIE 11 | Maintain capability |
| ZIE 12 | Improve capability |
| ZIE 13 | Restore capability |
| ZIE 14 | Support capability |
| ZIE 15 | Delegate / transfer tasks |
| ZIE 16 | Support organization |
| ZIE 17 | Delegate organization |
| ZIE 18 | Take over organization |
| ZIE 19 | Make information accessible |
| ZIE 20 | Provide information |
| ZIE 21 | Make existing aids usable |
| ZIE 22 | Acquire new aids and make them usable |
| ZIE 23 | Arrange living space according to needs |
| ZIE 24 | Create financial relief |
| ZIE 25 | Debt relief |
| ZIE 26 | Stabilize financial situation |
| ZIE 27 | Patient’s wishes can be expressed |
| ZIE 28 | Ensure patient’s will is implemented |
| ZIE 29 | Clarify legal representation |
| ZIE 30 | Clarification of legal issues |
| ZIE 31 | Make existing devices (e.g., tablet, smartphone) usable |
| ZIE 32 | Acquire and make new devices (e.g., tablet, smartphone) usable |
| ZIE 33 | Maintain existing social contacts |
| ZIE 34 | Deepen existing social contacts |
| ZIE 35 | Find new social contacts |
| ZIE 36 | Restore old social contacts |

B. Actions

B1. General Actions

| MAA 00 | Other general actions |
| --- | --- |
| MAA 01 | Supportive conversation |
| MAA 02 | Counseling |
| MAA 03 | Accompaniment |
| MAA 04 | Crisis intervention |
| MAA 05 | Providing information |
| MAA 06 | Obtaining information |
| MAA 07 | Pastoral care |

B2. Actions on organizational aspects

| MAO 00 | Other organizational actions |
| --- | --- |
| MAO 01 | Appointment scheduling |
| MAO 02 | Rescheduling appointment |
| MAO 03 | Appointment cancellation |
| MAO 04 | Establishing contact |
| MAO 05 | Forwarding information to cooperation partners |
| MAO 06 | Completing forms |
| MAO 07 | Support in filling out application forms |

B3. Actions on health

| MAG 00 | Other health actions |
| --- | --- |
| MAG 01 | Diagnostic clarification: neurological |
| MAG 02 | Diagnostic clarification: internal medicine |
| MAG 03 | Diagnostic clarification: urological |
| MAG 04 | Diagnostic clarification: gynecological |
| MAG 05 | Diagnostic clarification: psychiatric |
| MAG 06 | Diagnostic clarification: other specialty |
| MAG 07 | Palliative medical treatment |
| MAG 08 | Initiation of an interdisciplinary case discussion |
| MAG 09 | Pain therapy |
| MAG 10 | Clarification of medication-based treatment |
| MAG 11 | Guidance in handling immunotherapeutics |
| MAG 13 | Guidance in handling other medications |
| MAG 14 | Physiotherapy |
| MAG 15 | Speech therapy |
| MAG 16 | Ergotherapy |
| MAG 17 | Learning relaxation techniques |
| MAG 18 | Nutritional counseling |
| MAG 19 | Exercise / physical activity |
| MAG 20 | Support in finding suitable sports activities |
| MAG 21 | Monitor / observe consumption |
| MAG 22 | Organization of a nursing service for treatment care |
| MAG 23 | Organization of a palliative care service |
| MAG 24 | Organization of an SAPV team (specialized outpatient palliative care) |

B4. Actions on care and self-care

| MPS 00 | Other nursing and self-care actions |
| --- | --- |
| MPS 01 | Partial assistance with personal hygiene |
| MPS 02 | Full assistance with personal hygiene |
| MPS 03 | Hairdresser visit |
| MPS 04 | Foot care |
| MPS 05 | Full home cleaning |
| MPS 06 | Partial home cleaning |
| MPS 07 | Meal preparation |
| MPS 08 | Grocery shopping |

B.5 Actions on assistive devices and home modifications

| MHW 00 | Other actions relating to assistive devices / home modifications |
| --- | --- |
| MHW 01 | Nursing bed |
| MHW 02 | Alternating pressure mattress |
| MHW 03 | Positioning mattress |
| MHW 04 | Patient lift |
| MHW 05 | Toilet/shower chair |
| MHW 06 | Bedpan/urinal bottle |
| MHW 07 | Signal button/mat |
| MHW 08 | Eating aids (special cutlery, dishes, etc.) |
| MHW 09 | Modification of toilet or other sanitary facilities |
| MHW 10 | Raised toilet seat |
| MHW 11 | Armrests beside the toilet |
| MHW 12 | Bidet installation |
| MHW 13 | Wall-mounted grab bars |
| MHW 14 | Hygiene aids |
| MHW 15 | Manual wheelchair |
| MHW 16 | Customised electric wheelchair |
| MHW 17 | Walker / rollator |
| MHW 18 | Walking aids (e.g., crutches) |
| MHW 19 | Orthosis (e.g., peroneal splint) |
| MHW 20 | Bathroom modification (barrier-free) |
| MHW 21 | Special bathtub |
| MHW 22 | Mobile or bathtub-mounted patient lift |
| MHW 23 | Other transfer or lifting aids |
| MHW 24 | Handrails / grab bars |
| MHW 25 | Folding shower seat / shower stool |
| MHW 26 | Kitchen stove (e.g., height-adjustable) |
| MHW 27 | Kitchen cabinets |
| MHW 28 | Table, chairs |
| MHW 29 | Kitchen counter, sink area |

B.6 Actions on mobility and living

| MMW 00 | Other measures relating to mobility and living |
| --- | --- |
| MMW 01 | Procurement of individual means of transportation |
| MMW 02 | Modification of individual means of transportation |
| MMW 03 | Wheelchair training |
| MMW 04 | Information on barrier-free public transport options in the local area |
| MMW 05 | Contact with public transport companies |
| MMW 06 | Assistance with housing search |
| MMW 07 | Housing search |
| MMW 08 | Support in finding a care facility / hospice |
| MMW 09 | Organization of relocation |

B.7 Actions on social law, labour and financial aspects

| MSF 00 | Other actions relating to social law, labour and financial aspects |
| --- | --- |
| MSF 01 | Preparation of an advance healthcare directive |
| MSF 02 | Preparation of a power of attorney |
| MSF 03 | Application for legal guardianship |
| MSF 04 | Preparation of a will |
| MSF 05 | Clarification of account management |
| MSF 06 | Application for a disability ID card |
| MSF 07 | Preparation of a financial overview |
| MSF 08 | Pension application |
| MSF 09 | Application for social benefits |
| MSF 10 | Contacting health insurance providers |
| MSF 11 | Contacting long-term care insurance providers |
| MSF 12 | Contacting other social service providers |
| MSF 13 | Legal consultation |
| MSF 14 | Contacting employer(s) |
| MSF 15 | Involving employee representation / disability representatives |
| MSF 16 | Reduction of working hours |
| MSF 17 | Voluntary work |
| MSF 18 | Change of workplace |

B.8 Actions on social situation and participation

| MST 00 | Other actions relating to social situation and participation |
| --- | --- |
| MST 01 | Family counselling |
| MST 02 | Couple counselling |
| MST 03 | Family therapy |
| MST 04 | Couple therapy |
| MST 05 | Bereavement support |
| MST 06 | Information about local associations |
| MST 07 | Support in finding self-help groups |
| MST 08 | Peer support |
| MST 09 | Contact with family |
| MST 10 | Organization of family/friends gatherings |
| MST 11 | Procurement of computer, smartphone, tablet |
| MST 12 | Adaptation of electronic communication devices |
| MST 13 | Support in organizing vacation travel |
